# An Open-Source Deep Learning-Based GUI Toolbox For Automated Auditory Brainstem Response Analyses (ABRA)

**DOI:** 10.1101/2024.06.20.599815

**Published:** 2024-06-20

**Authors:** Abhijeeth Erra, Jeffrey Chen, Elena Chrysostomou, Shannon Barret, Cayla Miller, Yasmin M. Kassim, Rick A. Friedman, Federico Ceriani, Walter Marcotti, Cody Carroll, Uri Manor

**Affiliations:** 1 Data Institute, University of San Francisco, San Francisco, CA; 2 Dept. of Mathematics and Statistics, University of San Francisco, San Francisco, CA; 3 Dept. of Cell & Developmental Biology, University of California San Diego, La Jolla, CA,; 4 Dept. of Otolaryngology, University of California San Diego, La Jolla, CA; 5 Dept. of Biomedical Science, University of Sheffield, Sheffield, S10 2TN, UK.; 6 Neuroscience Institute, University of Sheffield, Sheffield, S10 2TN, UK.; 7 Halıcıoğlu Data Science Institute, University of California San Diego, La Jolla, CA

## Abstract

In this paper, we introduce a new, open-source software developed in Python for analyzing Auditory Brainstem Response (ABR) waveforms. ABRs are a far-field recording of synchronous neural activity generated by the auditory fibers in the ear in response to sound, and used to study acoustic neural information traveling along the ascending auditory pathway. Common ABR data analysis practices are subject to human interpretation and are labor-intensive, requiring manual annotations and visual estimation of hearing thresholds. The proposed new Auditory Brainstem Response Analyzer (ABRA) software is designed to facilitate the analysis of ABRs by supporting batch data import/export, waveform visualization, and statistical analysis. Techniques implemented in this software include algorithmic peak finding, threshold estimation, latency estimation, time warping for curve alignment, and 3D plotting of ABR waveforms over stimulus frequencies and decibels. The excellent performance on a large dataset of ABR collected from three labs in the field of hearing research that use different experimental recording settings illustrates the efficacy, flexibility, and wide utility of ABRA.

## Introduction

**Figure 1:**
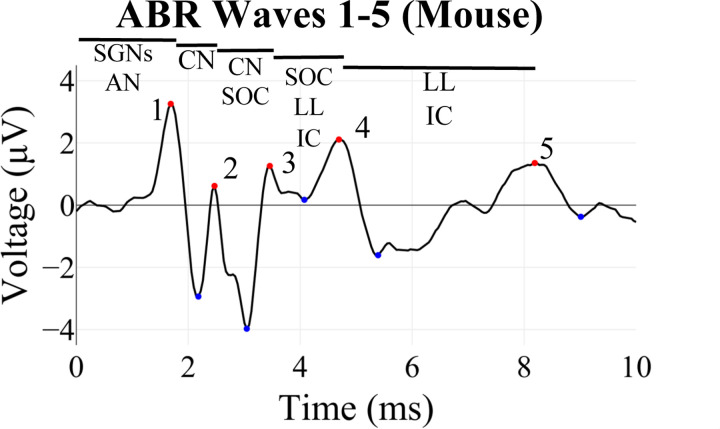
Example of an ABR waveform recorded from a mouse showing its characteristic features or waves. Wave 1 is generated by the spiral ganglion neurons (SGNs) and auditory nerve (AN), Wave 2 by the cochlear nucleus (CN), Wave 3 by the CN and superior olivary complex (SOC), Wave 4 by the SOC, lateral lemniscus (LL) and inferior colliculus (IC), and Wave 5 by the LL and IC (Rüttiger et. al. 2017, Melcher et al. 1996, Henry 1979, Land et al. 2016). Peaks of these waves are denoted by red dots, and troughs with blue dots.

Auditory brainstem response (ABR) recordings provide an objective measurement of electrical activity along the ascending auditory neural pathway, starting from the afferent fibers innervating the inner hair cells in the cochlea through the brainstem nuclei (Eggermont 2019; Kim et al. 2022; Burkard and Don 2012; Ingham et al. 2011; Møller and Jannetta 1985; Xie et al. 2018). ABRs are widely used in auditory research to study acoustic neural information transmission and to diagnose and distinguish different forms of hearing loss and synaptopathy in animal models of human otologic and neurologic conditions (Sininger 1993; Burkard and Sims 2001; Fernandez et al., 2015; Bramhall et al., 2018; Bao et al., 2022; Young, Cornejo, and Spinner 2023). In mice, ABR waveforms consist of five characteristic peaks (Figure 1), each approximately corresponding to the sound-induced electrical signal traveling through the different structures along the auditory pathway (Figure 1, Rüttiger et. al. 2017, Melcher et al. 1996, Henry 1979, Land et al. 2016).

A key goal of ABR threshold analysis in mice is to generate quantitative measures of hearing function, defined as the minimum sound intensity (in decibels) at a given frequency that elicits a repeatable neural response. Traditionally, threshold identification is performed by visually inspecting waveforms at decreasing sound intensities until a waveform is no longer distinguishable from baseline noise. ABR thresholds in anesthetized mice are typically ~10dB higher than behavioral perceptual responses in awake mice (Radziwon et al. 2009). While pragmatic, this method is time-consuming for larger studies and prone to inconsistency and bias between labs and examiners (Suthakar and Liberman 2019, Schrode 2022). To address these limitations, heuristic and machine learning computational approaches have been explored for automated ABR analysis. Supervised learning models (i.e. models which learn from data with ground truth labels) like convolutional neural networks (CNN), gradient boosting machines, and others have been used to accurately analyze suprathreshold ABR waveforms (Wimalarathna et al. 2021, McKearney and MacKinnon 2019, Kamerer et al. 2020) and to assess the degree of synaptopathy in humans (Buran et al. 2022). The utility of unsupervised learning models (i.e. models which learn from data without ground truth labels) for ABR analysis remains relatively unexplored to date. Assuming a similar amount of training data, unsupervised models often have a harder task than supervised models since they cannot learn from true labels. However, application of unsupervised methods often comes with reduced human labor requirements, since they do not require manually annotated ground truth data from which to learn.

In this paper, we introduce the Auditory Brainstem Response Analyzer (ABRA), a novel open-source software that implements a collection of supervised and unsupervised machine learning models trained on a diverse range of mouse ABR datasets from multiple labs for comprehensive and maximally generalizable mouse ABR analysis. ABRA is a user-friendly, browser-based application that supports batch data import/export, waveform visualization, automated peak detection, threshold estimation, latency quantification, time warping for curve alignment, and interactive 2D/3D plotting. By integrating these diverse functionalities into a unified platform, ABRA aims to streamline ABR data processing and analysis, reduce manual labor, and facilitate standardization and reproducibility across labs. We demonstrate ABRA’s flexibility and generalizability by benchmarking its performance on ABR datasets collected from three different hearing research labs using distinct experimental protocols and recording settings.

## Methods

### Data Collection

To test for the generalizability and flexibility of developed open-source ABR software, we used three distinct datasets from different labs to train and evaluate ABRA’s models (Tables 1 - 2). Each dataset used in the analysis was collected under unique experimental conditions and protocols. All three labs used a similar overarching methodology, including the use of anesthesia, electrodes, and sound decibel (dB) ranges. However, there were also differences in the specifics of these procedures, as outlined in Table 1. These differences underscore the flexibility of ABRA in accommodating diverse experimental setups and protocols. Further details on data collection conditions are available in the Supplementary Information.

### The ABRA Graphical User Interface

The proposed Auditory Brainstem Response Analyzer (ABRA) software was built to facilitate the examination and analysis of ABR waveforms. ABRA was developed in Python using the Streamlit framework (“Streamlit” n.d.) and provides an interactive platform for researchers to visualize ABR data. The app is hosted at https://ucsdabranalysis.streamlit.app/ and all documentation can be found on our Github: https://github.com/ucsdmanorlab/abranalysis. ABRA allows users to import multiple ABR data files stored in either .arf or .csv files obtained from BioSigRZ software from Tucker Davis Technologies (TDT). Upon import, the data is preprocessed to extract frequency, dB level, and the waveform data itself.

**Figure 2:**
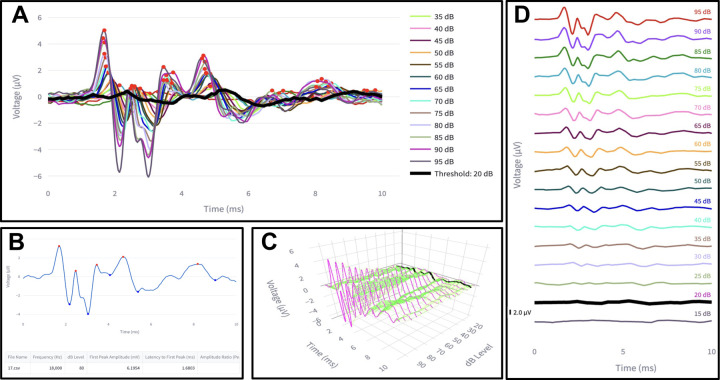
Screenshots from the ABRA app highlighting the different functionalities of ABRA **(A)** visualizing several ABR waveforms from one 1 mo male C57Bl/6N mouse across different togglable dBs at 18 kHz with predicted peak locations (red points) and predicted threshold (thick black line). **(B)** plotting a single ABR waveform at a specific sound frequency and intensity (dB SPL) with peaks and troughs labeled. **(C)** 3D plotting of all ABR waveforms at a given frequency with the predicted threshold (20dB) highlighted in black (can be rotated in the app). **(D)** stacks of ABR waveforms as a function of increasing dB SPL from the same frequency with the predicted threshold (20 dB) highlighted in black.

At its simplest functionality, ABRA allows the user to select which frequencies and decibel levels they wish to examine. The ABR plots are shown through the Plotly framework in Python and can be downloaded as .png files. ABRA displays metrics under the plots related to the displayed waveforms, including Wave 1 amplitude, and latency to the first peak. These metrics can be downloaded into a .csv file. ABRA also allows the user to view all the waveforms for a single frequency, highlights the automatically detected peaks and troughs, and automates thresholding so that analysis can be performed more efficiently (Figure 2).

For those seeking a comprehensive view of the variations in the waveform over several dB levels at the same frequency for thresholding, ABRA provides the option to implement time warping which registers the peaks of the waveforms of multiple dBs in response to the same frequency of stimulation (see Figure 5). The app also provides a 3D surface plot of waveforms which is interactive and allows the user to view the series of ABR waveforms as cross-sections of the ABR voltage surface over the decibel and time domains. ABRA’s different functionalities can provide the user the tools to visually threshold for themselves and compare their threshold with our model’s prediction. ABRA also allows users to conduct these analyses for multiple data files in batches at the same time.

### ABRA Peak Detection

**Figure 3:**
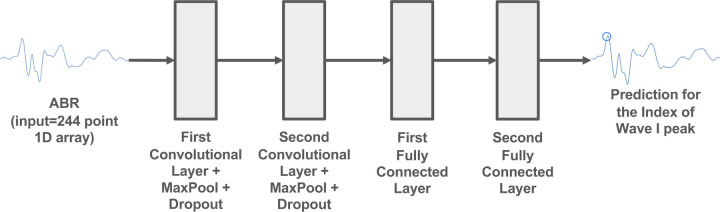
Model architecture for Wave 1 Peak Finding Algorithm. The ABR waveform recorded over 10 ms is input into two sequential layers of Convolution, Maxpool, and Dropout. The dimensionality of the output is reduced through two consecutive fully-connected layers which returns the prediction of the time point of the Wave 1 peak.

ABRA incorporates a two-step peak finding algorithm that leverages Pytorch’s deep learning library and the Scikit-learn library. The first step involves deploying a pre-trained Convolutional Neural Network (CNN) to retrieve a prediction for the location of the Wave 1 peak. We had 767 ABRs with ground truth labeled Wave 1 latencies and amplitudes (358 ABR waveforms from Lab A, 40 mice; 409 ABR waveforms from Lab B, 4 mice). We included 409 ABRs from Lab B to have a diversity of ABRs so that the model can be more thoroughly generalizable. Before training the CNN, the dataset was split into two sets with 80% of data from each lab going into the training set and 20% of data from each lab going into the testing set. The CNN was trained on 613 ABRs (286 ABRs from Lab A, 40 mice; 327 ABRs from Lab B, 4 mice) of length 244 (representing 10 ms) labeled with ground truth data related to the Wave 1 peak. The CNN optimizes squared error loss for the regression task which returns a prediction for the Wave 1 peak timepoint. A sparse representation of the network architecture is shown in Figure 3.

The CNN’s prediction of the Wave 1 peak time point serves as a reasonable initial estimate but ABRA further performs some fine-tuning in order to ensure that it is not sitting at a point neighboring the peak. To retrieve the correct point of the peak of Wave 1, a second fine-tuning step was implemented as follows. First, the ABR was smoothed using Gaussian smoothing to attenuate or remove nuisance peaks to identify peak indices. Then the *find_peaks* method from Scikit-learn was used to identify the remaining Wave 2–5 peak/trough locations and voltages by searching for all local maxima and minima by simple comparison of neighboring values of the wave starting from the CNN predicted Wave 1 peak index. Afterwards, the unsmoothed waveforms are utilized to quantify the amplitudes at the previously identified indices. The parameters for these methods were optimized using ground truth Wave 1 latency from 154 ABR waveforms (72 ABR waveforms from Lab A, 34 mice; and 82 ABR waveforms from Lab B, 4 mice) and ground truth Wave 4 amplitude from 211 labeled ABRs from Lab A. These parameters include the following:

- Window size for the start point for the smoothed waveform being inputted into the find_peaks function (optimized to 0.3689 ms before the CNN prediction for Wave 1 peak).
- Time between peaks such that the correct peaks are identified (optimized to 0.7377 ms).
- Bandwidth parameter for the Gaussian smoothing step was set to *σ* = 1.0.
- Time between troughs such that the correct troughs are identified (optimized to 0.5738 ms).

### Supervised Threshold Estimation with ABRA

The threshold estimation method used a binary machine learning classifier to identify individual ABR waveforms as either above or below threshold. Once individual waveforms were identified, the hearing threshold for a given frequency was determined as the quietest stimulus level (in dB SPL) for which a subject’s ABR waveform suggested a hearing response (i.e. was above threshold). Three candidate supervised binary classifiers were trained and evaluated: A CNN, an XGBoost classifier, and a Logistic Regression Classifier.

The dataset comprised 23,352 ABR waves from 221 mice (Lab A = 48 mice; Lab B = 104 mice; Lab C = 36 mice), with each wave characterized by its frequency, decibel level, and amplitudes at 244 uniformly distributed sampling points over a 10 ms time window. ABRs not initially sampled at 244 samples per 10 ms were resampled using linear interpolation. The ABRs were grouped by subject and frequency, then 80% of these groups were randomly allocated for training (Lab A = 34 mice; Lab B = 83 mice; Lab C = 27 mice) and the remaining 20% were designated for testing (Lab A = 14 mice; Lab B = 21 mice; Lab C = 9 mice). This method ensures a representative distribution of ABRs from various subjects and frequencies across the training and testing sets. Accordingly, the training input matrix had dimensions of 18,686 × 246, where 18,686 is the total number of training samples and 246 is the number of features, including 244 voltage recordings for each ABR, the decibel level, and the frequency of the stimulus.

**Figure 4:**
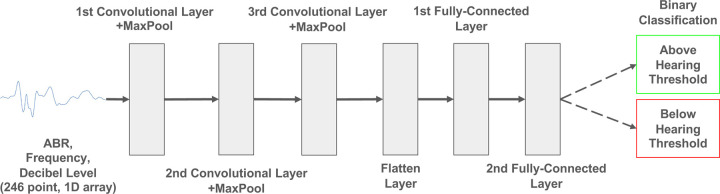
Model architecture for CNN ABR Classifier. The ABR waveform recorded over 10 ms and its frequency and decibel level are input into three sequential layers of convolution and max pooling. The dimensionality of the output is reduced through two consecutive fully-connected layers before returning the classification.

**Figure 5:**
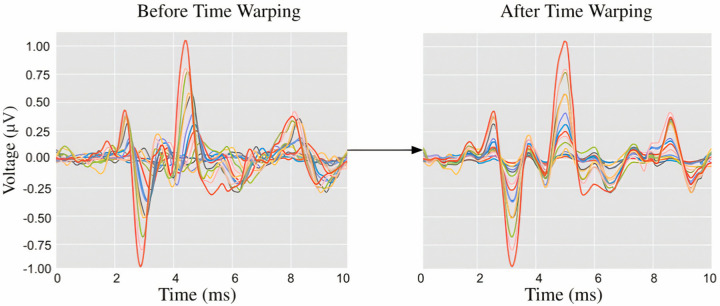
ABRs before (left) and after (right) Time Warping. The depicted transformation of waveforms, both before and after applying elastic time warping using the *fdasrsf* package (Tucker 2020), illustrates clear registration of waveform features. Associated with each waveform is also an estimated time warping function which is useful in quantifying changes between the original unaligned latencies to the aligned latencies for all wave peaks and troughs.

For the Logistic Regression Classifier and XGBoost Classifier, time warping was used on the ABR trajectories as an additional preprocessing step to align waveform features such as peaks and troughs (see section below: ABR Curve Alignment with Time Warping). For the CNN, no additional preprocessing steps were used. The architecture of the CNN is described in Figure 4.

### ABR Curve Alignment with Time Warping

As previously discussed, ABRs from mice often exhibit a characteristic structure with 5 distinct peaks (Figure 1). However, a common challenge in analyzing these ABR waveforms is the non-uniform latency across different frequencies and decibel levels. This variability in latency can distort the time-based comparison of these responses, as the peaks do not occur at the same time instances across different ABRs. To address this, we provide an option to employ time warping to align these ABRs, which standardizes the position of peaks and other salient features of the ABRs across time. This alignment serves dual purposes. First, it decouples amplitude and phase variation, facilitating the visual comparison of amplitudes of ABR waveforms. Second, the encoding of time alignment parameters into individual-specific warping functions provides the option of incorporating these features into machine learning models, which in some cases improves the models’ performance and predictive power. It is important to emphasize that the optional time warping as a preprocessing step should only be used when analyzing amplitude, but not latency variability.

To conduct the time warping step, we used the *fdasrsf* package in Python (Tucker 2020). This package implements elastic time warping, a method that allows for alignment of key signals in waveforms. This technique is particularly useful in our case, as it allows us to align the ABRs despite the non-uniform latency across different frequencies and decibel levels.

### Unsupervised Threshold Estimation

ABRA also provides an optional method to implement an unsupervised ABR threshold estimation, which uses ABR waveforms at a specific frequency across multiple dB levels. Following the optional time alignment of waveforms (see above section: ABR Curve Alignment with Time Warping), Functional Principal Component Analysis (FPCA) (Kleffe 1973) is used to identify and quantify what an eigenanalysis determines to be the most significant patterns of variation in the ABR waveforms (e.g. averages and contrasts in wave peaks, troughs, and amplitudes, etc.). The waveforms are then projected onto their first and second principal components (PCs). This projection serves to reduce dimension, separate signal from noise, and cluster waveforms with similar salient ABR features, thereby simplifying the high-dimensional waveform data into a simple 2-dimensional representation. Only the first two PCs were used because they captured 95% of the variance in a set of typical ABRs, which indicates most of the signal can be represented using just these two components. Truncating at two components also discards later components which tend to be associated with noise.

Finally, ABRA employs a k-means clustering algorithm with 2 clusters on the projected data. The underlying assumption is that ABR waveforms above hearing threshold have higher principal component scores and will be clustered together, while the ABR waveforms below hearing threshold will form a separate cluster of near-zero principal component scores. This unsupervised approach allows users to identify natural groupings in the data without any prior assumptions about the number or characteristics of these groups.

## Results

### Peak Amplitude and Latency Estimation

To benchmark ABRA’s performance in peak amplitude and latency estimation, we fed a test set of 154 ABRs with human-labeled “ground truth” Wave 1 amplitude and latency values from Lab A (72 waveforms from 34 mice) and Lab B (82 waveforms from 4 mice) into ABRA. The ground truth values for Lab A data were obtained by using visual examination from two observers, while the ground truth values for Lab B data were obtained from manual labeling using custom software. Though it is possible to make manual adjustments to ABRA, we compare here only the absolute differences resulting from the automated (i.e. unadjusted) estimates generated from ABRA vs their corresponding human-labeled ground truth values in order to fairly assess its underlying model.

For each ABR waveform in the sample, let $\tau_{i}^{\left(GT\right)}$ denote the corresponding ground truth latency and let $a_{i}^{\left(GT\right)}$ denote the corresponding ground truth amplitude, with waveforms indexed by i=1,…,n. Then, let $\tau_{i}^{\left(ABRA\right)}$ denote the Wave 1 latency estimates generated by each software; similarly let $a_{i}^{\left(ABRA\right)}$ denote ABRA’s generated Wave 1 amplitude estimates.

Errors are then defined as the differences between a given software’s estimate and the ground truth value. We define errors for Wave 1 latencies and amplitudes, respectively, as follows:

$$e_{\tau ,i}=\tau_{i}^{\left(ABRA\right)}-\tau_{i}^{\left(GT\right)},i=1,\ldots ,n,and$$

$$e_{a,i}=a_{i}^{\left(ABRA\right)}-a_{i}^{\left(GT\right)},i=1,\ldots ,n.$$

**Figure 6:**
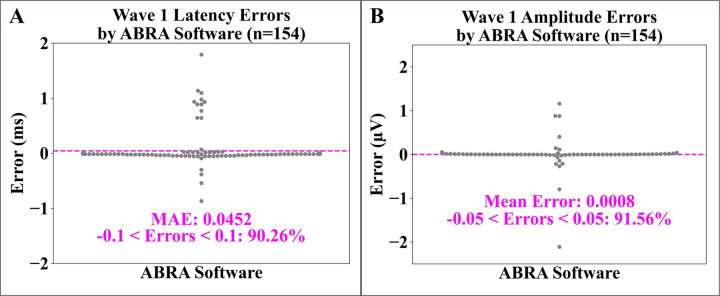
Swarmplots displaying spreads of error for detected Wave 1 Latency (A) and Amplitude (B) vs. ground truth for each software. Testing failed to find evidence that mean absolute errors were significantly greater than zero for both Wave 1 Latency and Amplitude estimates. 90.26% of all ABRA-generated estimates of Wave 1 Latency were within 0.1ms of the corresponding true human-labeled latency; 91.56% of all ABRA-generated Wave 1 amplitude were within 0.05 µV of the corresponding true human-labeled peak amplitude. n = 154 represents the number of ABRs tested. Related statistics are listed in **Table 3.**

Side-by-side swarmplots of the distributions for error are shown for latency and amplitudes in Figure 6A and 6B, respectively; summary statistics for errors are displayed in Table 3.

Testing whether the centers of these distributions differed from zero showed that the average Wave 1 Latency errors produced by ABRA did not detect a significant deviation from zero (${\underline{e}}_{\tau }=0.0452$, *SE = 0.0230, p = 0.0512)* which suggests that ABRA is on average closely aligned with the human-labeled ground truth latencies. In a parallel comparison for amplitude estimates, hypothesis testing on the Wave 1 amplitude error distributions found that the average Wave 1 Amplitude error produced by ABRA did not deviate significantly from zero (${\underline{e}}_{a}=-0.015$, *SE = 0.0192, p = 0.9357)* which suggests that ABRA amplitude estimates are on average closely aligned with the human-labeled ground truth amplitudes.

**Figure 7:**
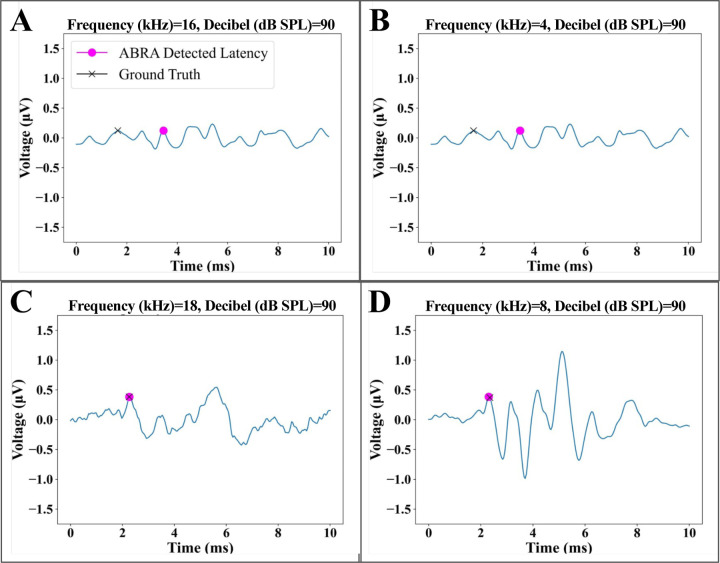
Examples of error cases in peak detection. **(A)** and **(B)** display examples of multiple peaks that may be identified as Wave 1 by different softwares and different sets of eyes, which are more difficult for ABRA to correctly detect. **(C)** and **(D)** are examples of ABR waveforms with larger signal to noise ratios for which ABRA matches the ground truth.

These comparisons show that ABRA-generated estimates generally agree with human-labeled ground truth latency and amplitude estimates, and when adjustments are needed they are small in magnitude. Figure 7 displays a few visual examples of how errors from the ABRA software may arise, with the most common source of errors arising from ABRs with very low signal-to-noise ratios (SNR).

### ABR Classification and Threshold Estimation Results

**Figure 8:**
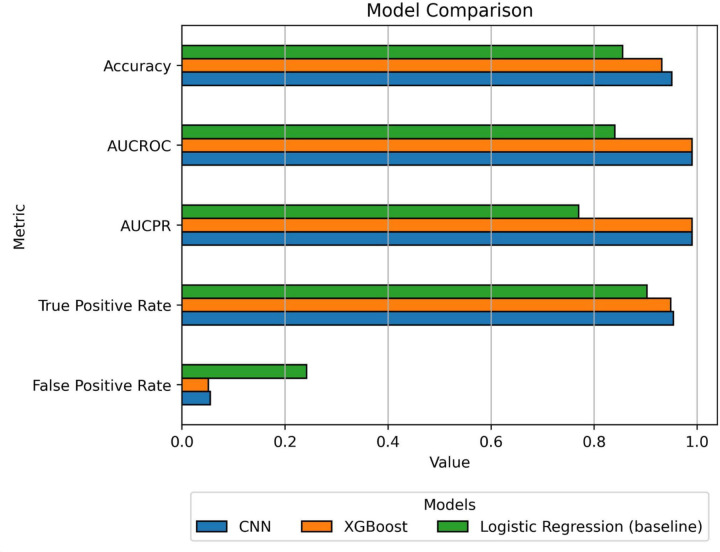
Comparative Analysis of Machine Learning Models. Horizontal bar chart illustrating the performance of three machine learning models: Convolutional Neural Network (CNN), XGBoost, and Logistic Regression (baseline). The metrics used for comparison are Accuracy, True Positive Rate, False Positive Rate, Area Under the Receiver Operating Characteristic Curve (AUCROC), and Area Under the Precision-Recall Curve (AUCPR). The CNN model exhibits the highest accuracy, while the Logistic Regression model serves as the baseline for comparison. Related statistics are listed in Table 4.

**Figure 9:**
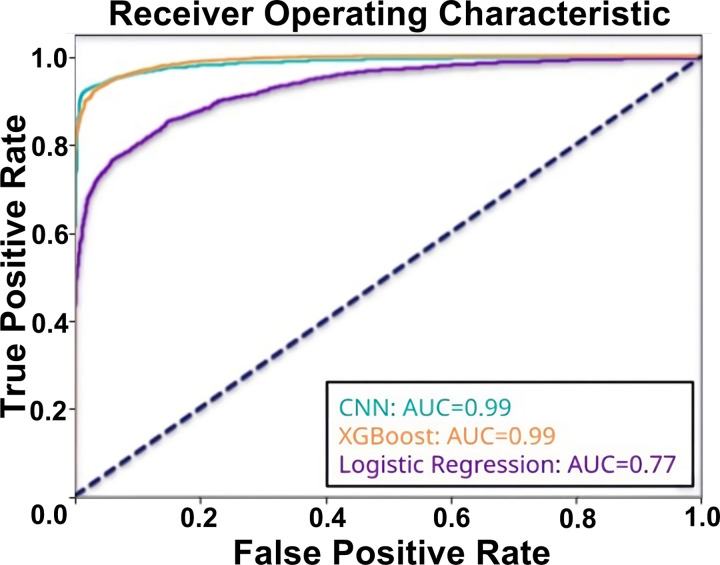
Receiver Operating Characteristic (ROC) Curves and Areas Under Curves (AUC) for Convolutional Neural Network (CNN), XGBoost, and Logistic Regression Classifiers. A ROC curve demonstrates the performance of an ABR classifier at all classification thresholds. The area under the ROC curve represents the ABR classifier’s overall ability to distinguish between ABR responses that are above the hearing threshold and those that are not under varying model settings. The ROC curves for the CNN and XGBoost classifiers are nearly identical, while the Logistic Regression classifier shows relatively inferior performance.

The performance of our ABR classifiers for threshold detection was assessed on the testing set of 5,384 ABR waveforms. Performance metrics are shown in Figure 8 and a pairwise comparison for significance is provided in Table 4. As a simple and interpretable model, logistic regression was used as a baseline for the binary classification task. Despite its simplicity, it achieved an accuracy of 85.56%, a True Positive Rate (TPR), sometimes referred to as recall or sensitivity, of 90.27%, and an Area Under the Receiver Operating Characteristic Curve (AUROC) of 0.84. However, its performance was significantly outperformed by both the CNN and XGBoost models. The CNN model demonstrates superior performance in terms of accuracy (95.08%) and TPR (95.36%). These metrics surpass those of both the XGBoost and the baseline Logistic Regression models, indicating the CNN’s enhanced ability in correctly identifying ABR thresholds. However, it is noteworthy that the XGBoost model exhibits a slightly lower False Positive Rate (FPR) of 5.10%, compared to the CNN model’s 5.49%. This suggests that the XGBoost model may be more effective in reducing false positives. Both the CNN and XGBoost models achieved similar AUROC and Area Under the Precision-Recall Curve (AUPRC) of 0.99 (Figure 9). These metrics indicate promising sensitivity and precision.

**Figure 10:**
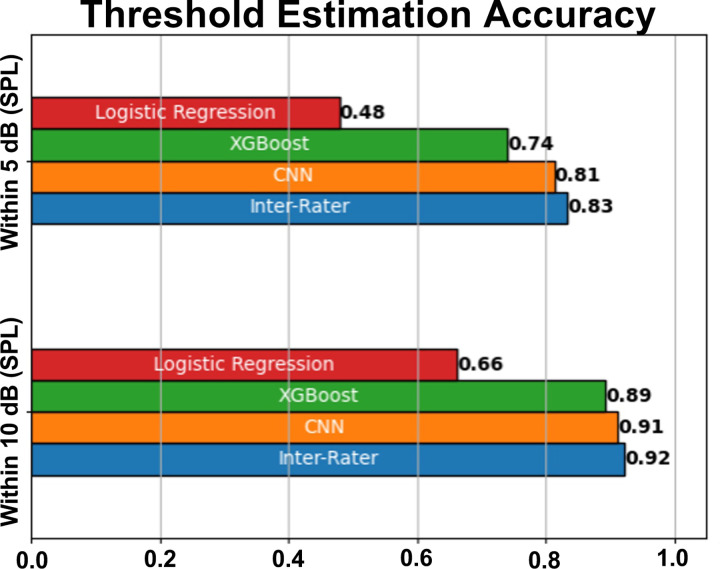
Threshold Estimation Metrics for Machine Learning Models and Human Expert Inter-Rater Comparison (statistics in Table 5). Bar chart comparing the performance of three machine learning models: Convolutional Neural Network (CNN), XGBoost, and Logistic Regression, as well as an Inter-Rater comparison based on their ability to estimate thresholds within 5dB SPL and 10dB SPL. The Inter-Rater comparison was conducted by comparing 100 threshold estimates of two experts. The CNN and XGBoost models demonstrate superior performance compared to the Logistic Regression model, with the CNN outperforming XGBoost at higher levels of precision. The Inter-Rater comparison provides a benchmark for human expert performance in the task.

In Figure 10, we compare the performance of three classifiers: Convolutional Neural Network (CNN), XGBoost, and Logistic Regression. We also include an Inter-Rater comparison, which reflects the proportion of ABRs for which two experts agree on a threshold within some envelope (within 5dB SPL, 10dB SPL, etc.), providing a real-world benchmark for performance.

The classifiers are evaluated based on their ability to estimate hearing thresholds, defined as the minimum sound intensity (in decibels) at a given frequency that elicits a repeatable neural response. The evaluation accuracy considers how frequently the estimated threshold falls within 5dBs or 10dBs of the single-rater ground truth threshold. Table 5 provides inferential statistics for the differences between each model and inter-rater assessment at both accuracy envelopes. The CNN was the only model which performed comparably to human-rater assessment at the 10dB accuracy window; however no model could reach such accuracy at the 5dB envelope.

The CNN demonstrates superior performance across both metrics compared with the Logistic Regression and XGBoost models. Furthermore, the temporal nature of the data is well-suited to the architecture of CNNs. Unlike traditional machine learning models, CNNs can effectively capture temporal dependencies in the data, which is crucial for tasks involving time-series data, such as our case of audio signal processing. The CNN model’s superior performance at the 5dB and 10dB envelopes along with its ability to handle the temporal nature of the data makes it the optimal choice for this task. Moreover, the CNN achieves similar performance as the Inter-Rater comparison, indicating that its ability to estimate hearing thresholds is on par with the consensus of two human experts using standard visual threshold inspection methods. This suggests that the CNN model can function as a reliable tool for estimating hearing thresholds, providing a machine learning-based approach that matches human expert performance.

The performance of our threshold estimation technique was compared against that of EPL-ABR (Suthakar & Liberman 2019) on a separate dataset of ABR waveforms from Lab C (Table 6). This smaller set of ABR waveforms (N = 292) was selected because EPL-ABR’s threshold estimation software requires data in the custom ABR file format used by the Eaton Peabody Laboratories. Our CNN method outperforms EPL-ABR’s threshold estimation method across all metrics except for FPR. One benefit of CNN is that it can be continuously trained and improved as more data is made available.

### Time Cost Analysis

In order to quantify the time savings of using ABRA, we sent a random sample of 10 ABR files from Lab B to two ABR raters from Lab A with a total of 90 frequencies to be analyzed. It took both raters approximately 1 hour to manually analyze the ABR thresholds. However, using ABRA, it took about 48 seconds to output the automated thresholds for each frequency, corresponding to 75x increased efficiency. The automated thresholds were within 5 dBs of Lab A inspection 73% of the time, 10 dBs 88% of the time, and 15 dBs 96% of the time. For comparison, inter-rater assessment showed that a Lab A annotator was within 5 dB of a Lab B annotator’s result 89% of the time, 10 dB 99% of the time, and 15 dB 100% of the time.

## Discussion

The aim of this study is to illustrate ABRA’s versatility and additional benefits compared to other available software such as ABRWAS and EPL-ABR (see Table 7). ABRA has been designed to be a multi-purpose and versatile software with extended functionality and to be able to handle input data acquired from different mouse strains, different laboratories, and recorded in different formats, including the widely used standard .arf files from BioSigRZ Tucker Davis Technology recordings, or .csv files from any number of other systems given limited rules related to file structure.

The tests done in the Manor lab have shown that ABRA’s automated thresholding method reduces the time costs of thresholding analyses by more than 50x, and can help streamline the process of extracting ABR thresholds from multiple subjects. In addition, the results can be exported to a .csv file for post-processing by the experimenter, and plots can be directly exported for publication if desired.

The deep learning techniques used in ABRA have some precedence not only in previous ABR studies but in the field of electrophysiology in general. A recent study showed that convolutional neural networks and long short-term memory architecture can automatically detect discrete records of protein movement (Celik et. al. 2020). Another electrophysiological study introduced a deep learning algorithm that infers latent dynamics from single-trial neural spiking data (Pandarinath et. al. 2018). This study used data from non-overlapping recording sessions that improved the inference of their model, similar to how our software accounts for a range of data collection protocols for improved generalizability. Both studies were designed to automate otherwise laborious and arduous tasks and simplify them such that future studies can be more accurate, more reproducible, and less time-consuming. The deep learning techniques used in our software have similar potential for ABR studies by streamlining the onerous task of labeling peaks and identifying thresholds. We envision future ABR acquisition protocols that can be guided by our software to avoid acquiring excess measurements after a threshold is reached.

While we have argued that ABRA is a powerful tool for ABR analysis, it is necessary to remark that it also has its limitations and there exist areas for future improvements. Currently ABRA can handle .csv and BioSigRZ .arf file inputs reliably, however functionality for analysis of BioSigRP .arf files is still in development. Calculation of amplitude/latency results and performing visualization when batch processing large numbers of ABR files (N>100 waveforms) may take several minutes, especially if the user chooses to implement the time warping functionality. ABRA developers are currently investigating the feasibility of moving computation to cloud-hosted GPUs to accelerate data processing. Similarly, quality of life improvements for manual relabeling of ABRA-generated peaks, latencies, and thresholds are also an area for future work. As for model limitations, the CNN-based thresholding model was trained only on mouse ABRs which had step sizes no larger than 20 dB SPL. Moreover, it was validated for automated amplitude and latency measurements for only Wave 1, leaving the remaining Waves 2–5 currently unvalidated, which can be pursued in future efforts as the model continues to incorporate new data from our labs and others. Most importantly, the accuracy of peak and threshold detection may not yet match that of the most seasoned experts in visual ABR analysis. While the time saved by automation may still yet be a worthwhile tradeoff for certain applications, an additional benefit is the deterministic nature of the model and therefore high reproducibility. Most importantly, we anticipate significant improvements in performance as the amount of training data is increased over time.

Overall, this study has shown that the ABRA interface is a flexible one-stop-shop software for ABR amplitude/latency estimation, thresholding, visualization, and all plots can be exported for generating figures. ABRA’s ease-of-use, generalizability, and diverse functionality serve as potential outlets to streamline data processing and resulting studies involving ABR analysis.

## Supplementary Material

Supplement 1

## Figures and Tables

**Table 1:** Summary of the experimental recording conditions used by the three labs. The datasets are described in the following order: Anesthesia, Preparation, Electrode Placement, Sound Stimuli, Recording, Sound Intensity, and Distance of the Speaker. The specific methods employed by each lab—Manor Lab (Lab A), Marcotti Lab (Lab B), Liberman Lab (Lab C)—are detailed in the supplementary information section.

Methods	Lab A	Lab B	Lab C
**Anesthesia**	Ketamine (90 mg/kg) + Xylazine (10 mg/kg)	Ketamine (100 mg/kg) + Xylazine (10 mg/kg)	Ketamine (100 mg/kg) + Xylazine (10 mg/kg)
**Environme nt**	Soundproof chamber, heating pad (37°C)	Soundproof chamber, heating pad (37°C)	Soundproof chamber, heating pad (37°C)
**Electrode Placement**	Subcutaneous recording electrode at vertex, reference behind right pinna, ground on left leg	Subdermal electrodes behind pinna (reference and ground), vertex (active)	Needle electrodes: vertex to ipsilateral pinna (recording), ground near tail
**Sound Stimuli**	5-ms tone pips (0.5 ms cos2 rise-fall), 21/sec	5-ms pips (1.0-ms rise-fall with cos2 onset envelope), 42.6/sec	5-ms pips (0.5-ms rise-fall with cos2 onset envelope), 30/sec
**Recording**	Filtered (300 Hz - 3 kHz), averaged using BioSigRZ software, 512 responses averaged	Customized software (Ingham et al., 2011), RZ6 auditory processor, 256 responses averaged	Amplified (10,000X), filtered (100 Hz - 3 kHz), averaged with A-D board in LabVIEW system, 1024 responses averaged
**Sound Intensity**	Decreased from 90 dB SPL to 10/20 dB SPL in 5 dB steps	0–95 dB SPL in 5 dB steps	Raised from ~10 dB below threshold to 80 dB SPL in 5 dB steps
**Speaker distance**	Open-field - 10 cm from ear	Open-field - 10 cm from ear	Closed-field - ~3 cm from the eardrum
**Mouse age/strains used**	3-month SAMP8 (Senescence-Accelerated Mouse-Prone 8) (Takeda et al. 1981)	1-month C57Bl/6N with and without corrected CDH23	7-week C57Bl/6J

**Table 2:** Breakdown of mouse and ABR waveform data by lab, model, and train and test splits. Figures and Tables relevant to a given dataset are enumerated in brackets in the last row.

Lab/Model	Peak Detection		Automatic Thresholding		
	Training Data	Test Data	Training Data	Test Data	ABRA vs. EPL-ABR
**Lab A**	40 mice (286 ABRs)	34 mice (72 ABRs)	65 mice (5,419 ABRs)	16 mice (2,031 ABRs)	–
**Lab B**	4 mice (327 ABRs)	4 mice (82 ABRs)	83 mice (12,948 ABRs)	21 mice (3,276 ABRs)	–
**Lab C**	–	–	29 mice (319 ABRs)	7 mice (77 ABRs)	27 mice (292 ABRs)
**Total [Relevant Figures & Tables]**	**44 mice** **(613 ABRs)** **[Fig. 3]**	**38 mice** **(154 ABRs)** **[Fig. 6; Table 3]**	**177 mice** **(18,686 ABRs)** **[Fig. 4]**	**44 mice** **(5,384 ABRs)** **[Figs. 8–10; Tables 4, 5]**	**27 mice** **(292 ABRs)** **[Table 6]**

**Table 3 (related to Figure 6):** Table showing the Mean Error Difference and their Standard Errors between ABRA-detected Wave 1 Latency and Amplitude and corresponding ground truth values detected by human reviewers. Testing failed to find evidence that mean error differences were significant for both Wave 1 Latency and Amplitude estimates. The peak finding method seems to be better for Wave 1 estimates in Lab B data (84 ABRs), but overall (154 ABRs) the errors are very low.

	Wave 1 Latency	Wave 1 Amplitude
**Mean Difference (± S.E.M.)**	ABRA vs. Ground Truth (ms)	ABRA vs. Ground Truth (µV)
Lab A (n_waveforms_=72, n_mice_=34)	0.0978 (±0.0467)	0.0008 (±0.0405)
Lab B (n_waveforms_=84, n_mice_=4)	-0.0010 (±0.0117)	-0.0036 (± 0.0071)
**Overall Test Set**	**0.0452 (±0.0230)**	**-0.0015 (±0.0192)**

**Table 4:** Comparative analysis of performance metrics between the Convolutional Neural Network (CNN), XGBoost (XGB), and Logistic Regression (LR) models (related to Figure 8). The CNN model shows comparable performance to the XGB model across all metrics, except accuracy for which it outperforms. Both CNN and XGB show significantly better performance than the LR model across most metrics. The p-values indicate the statistical significance of these differences, with smaller values indicating stronger evidence of a difference. Significance level notation after applying Bonferroni correction for multiple testing: 0.05 (*), 0.01 (**), 0.001(***).

Metric	Comparison	Difference Estimate	95% CIs for differences	*p-value*	Significance
**Accuracy**	CNN vs. XGB	0.0196	(0.0107, 0.0285)	1.59×10^−5^	***
	CNN vs. LR	0.0952	(0.0842, 0.1062)	1.21×10^−62^ (~0)	***
	XGB vs. LR	0.0756	(0.0640, 0.0872)	5.18×10^−37^ (~0)	***
**AUCROC** Area Under the Receiver Operating Characteristic Curve	CNN vs. XGB	0.0000	(−0.0038, 0.0038)	1.00	**NS**
	CNN vs. LR	0.1500	(0.1399, 0.1601)	2.21×10^−171^ (~0)	***
	XGB vs. LR	0.1500	(0.2452, 0.2748)	2.21×10^−171^ (~0)	***
**AUCPR** (Area Under the Precision-Recall Curve)	CNN vs. XGB	0.0000	(−0.0038, 0.0038)	1.00	**NS**
	CNN vs. LR	0.2200	(0.2084, 0.2316)	2.71×10^−270^ (~0)	***
	XGB vs. LR	0.2200	(0.2084, 0.2316)	2.71×10^−270^ (~0)	***
**TPR** (True Positive Rate)	CNN vs. XGB	0.0051	(−0.0031, 0.0133)	0.22	**NS**
	CNN vs. LR	0.0510	(0.0412, 0.0606)	1.51×10^−24^ (~0)	***
	XGB vs. LR	0.0458	(0.0359, 0.0557)	1.36×10^−19^ (~0)	***
**FPR** (False Positive Rate)	CNN vs. XGB	0.0039	(−0.0046, 0.0124)	.367	**NS**
	CNN vs. LR	-0.1870	(−0.2000, −0.1740)	5.20×10^−164^ (~0)	***
	XGB vs. LR	-0.1909	(−0.2038, −0.1780)	1.08×10^−172^ (~0)	***

**Table 5:** Inference for differences in accuracy between inter-rater accuracy and the Convolutional Neural Network (CNN), XGBoost (XGB), and Logistic Regression (LR) models (related to Figure 10). Within both the 5dB and 10dB envelopes, no significant difference between the CNN and XGB models and baseline inter-rater accuracy was detected, suggesting CNN and XGB are performing at a comparable level as a human reviewer; however LR did show significantly worse performance than inter-rater assessment. Significance level notation after applying Bonferroni correction for multiple testing: 0.05 (*), 0.01 (**), 0.001(***).

Accuracy Envelope	Comparison	Accuracy Difference	95% CIs for Accuracy Difference	*p-value*	Significance
**Within 5dB SPL**	CNN vs. IRR	0.02	(−0.0852, 0.1252)	0.7062	**NS**
	XGB vs. IRR	0.09	(−0.0154, 0.1955)	0.0545	**NS**
	LR vs. IRR	0.35	(0.2443, 0.4558)	7.992×10^−12^	***
**Within 10dB SPL**	CNN vs. IRR	0.01	(−0.0673, 0.0874)	0.8628	**NS**
	XGB vs. IRR	0.03	(−0.0476, 0.1075)	0.4300	**NS**
	LR vs. IRR	0.26	(0.1814, 0.3387)	8.718×10^−8^	***

**Table 6:** Performance Comparison of Threshold Estimation Algorithms on Lab C data only (292 ABR waveforms from 27 mice). This table presents a side-by-side comparison of two threshold estimation algorithms: EPL-ABR and ABRA. The metrics used for comparison include Accuracy, True Positive Rate, False Positive Rate, and the ability to estimate thresholds within 5dB, 10dB, and 15dB. The values are presented as mean (± standard error). ABRA demonstrates superior performance (bolded) in terms of accuracy and estimating thresholds within 5dB, while EPL-ABR has a higher True Positive Rate.

Metric \ Software	EPL-ABR	ABRA
**Accuracy**	93.12 (±1.476)%	**93.42 (±1.446)%**
**True Positive Rate**	**97.10 (±0.978)%**	90.00 (±1.749)%
**False Positive Rate**	14.52 (±2.055)%	**0.00 (±0.0)%**
**Within 5dB SPL**	57.14 (±2.886)%	**91.42 (±1.633)%**
**Within 10dB SPL**	91.43 (±1.633)%	**94.29 (±1.353)%**
**Within 15dB SPL**	**94.29 (±1.353)%**	**94.29 (±1.353)%**

**Table 7:** Comparison of software features/capabilities. Functionality and aspects of ABRA, the Auditory Brainstem Response Waveform Analyzer (ABRWAS) (Burke et al. 2023), and the EPL-ABR Peak Analysis App (Suthakar and Liberman 2019).

FEATURES	ABRA	ABRWAS	EPL-ABR
**Threshold Detection**	Automated thresholding with both supervised/unsupervised machine learning methods	No automated threshold estimation	Automated thresholding using cross-covariance calculations
**Peak Detection**	Automates peak and trough detection	Suggests peak and trough detection as a guide for human revision	Automates peak and trough detection and allows for human revision
**Time Warping**	Performs elastic time warping	No time warping	No time warping
**Batch Processing**	Supports batch processing	Supports batch processing	No batch processing
**Data Extraction**	Generate peaks, troughs, and a metrics table with a single click	Generates peaks and troughs with option to manually adjust labels	Generates peaks and troughs with option to manually adjust labels
**Metric Exports**	Metrics table only includes three waveform metrics and the threshold for each frequency	Comprehensive metrics table	Comprehensive metrics table
**Accessibility**	Free and open source	Free and open source	Free and open source
**Image Exports**	Can export .png and .pdf files	No functionality	No functionality
**Stability**	When errors arise, app can recover easily	Errors require software relaunch	When errors arise, app can recover easily
**File Type Support**	Accepts .arfs and .csvs; only a couple rules related to the file structure	Each file must follow the same structure	Only supports EPL file type
**Operating System**	Windows/Mac/Linux	Windows	Windows/Mac
**Web Support**	Web-based application that can also run locally	Run on local machines only	Run on local machines only

## References

[R1] Bao Jianxin, Jegede Segun Light, Hawks John W., Dade Bethany, Guan Qiang, Middaugh Samantha, Qiu Ziyu, Levina Anna, and Tsai Tsung-Heng. “Detecting Cochlear Synaptopathy through Curvature Quantification of the Auditory Brainstem Response.” Frontiers in Cellular Neuroscience 16 (2022). 10.3389/fncel.2022.851500. PMC8959412 35356798

[R2] Buran Brad. “Auditory Wave Analysis”. Zenodo, 2015. 10.5281/zenodo.17365.

[R3] Buran Brad N., McMillan Garnett P., Keshishzadeh Sarineh, Verhulst Sarah, and Bramhall Naomi F.. “Predicting synapse counts in living humans by combining computational models with auditory physiology.” The Journal of the Acoustical Society of America 151, no. 1 (2022): 561–576. 35105019 10.1121/10.0009238 PMC8800592

[R4] Burkard Robert, and Don Manny. “The auditory brainstem response (ABR).” Translational Perspectives in Auditory Neuroscience: Hearing Across the Life Span–Assessment and Disorders. San Diego, CA: Plural Publishing (2012): 161–200.

[R5] Bramhall Naomi F., McMillan Garnett P., Kujawa Sharon G., and Konrad-Martin Dawn. “Use of non-invasive measures to predict cochlear synapse counts.” Hearing research 370 (2018): 113–119. 30366194 10.1016/j.heares.2018.10.006 PMC7161203

[R6] Burke Kali, Burke Matthew, and Lauer Amanda M.. “Auditory brainstem response (ABR) waveform analysis program.” MethodsX 11 (2023): 102414. 10.1016/j.mex.2023.102414. 37846351 PMC10577057

[R7] Celik Numan, O’Brien Fiona, Brennan Sean, Rainbow Richard D., Dart Caroline, Zheng Yalin, Coenen Frans, and Barrett-Jolley Richard. “Deep-Channel uses deep neural networks to detect single-molecule events from patch-clamp data.” Communications Biology 3, no. 1 (2020): 3. 31925311 10.1038/s42003-019-0729-3 PMC6946689

[R8] Eggermont Jos J. “Auditory brainstem response.” Handbook of clinical neurology 160 (2019): 451–464. 31277868 10.1016/B978-0-444-64032-1.00030-8

[R9] Fernandez Katharine A., Jeffers Penelope WC, Lall Kumud, Liberman M. Charles, and Kujawa Sharon G.. “Aging after noise exposure: acceleration of cochlear synaptopathy in ‘recovered’ ears.” Journal of Neuroscience 35, no. 19 (2015): 7509–7520. 10.1523/jneurosci.5138-14.2015. 25972177 PMC4429155

[R10] Henry Kenneth R. “Auditory brainstem volume-conducted responses: origins in the laboratory mouse.” Ear and Hearing 4, no. 5 (1979): 173–178. 511644

[R11] Ingham Neil J., Pearson Selina, and Steel Karen P.. “Using the auditory brainstem response (ABR) to determine sensitivity of hearing in mutant mice.” Current Protocols in Mouse Biology 1, no. 2 (2011): 279–287. 26069055 10.1002/9780470942390.mo110059

[R12] Kamerer Aryn M., Neely Stephen T., and Rasetshwane Daniel M.. “A model of auditory brainstem response wave I morphology.” The Journal of the Acoustical Society of America 147, no. 1 (2020): 25–31. 32006985 10.1121/10.0000493 PMC7043862

[R13] Kim Ye-Hyun, Schrode Katrina M., and Lauer Amanda M.. “Auditory brainstem response (ABR) measurements in small mammals.” Developmental, physiological, and functional neurobiology of the inner ear (2022): 357–375.

[R14] Kleffe Jürgen. “Principal components of random variables with values in a seperable hilbert space.” Mathematische Operationsforschung und Statistik 4, no. 5 (1973): 391–406.

[R15] Land Rüdiger, Burghard Alice, and Kral Andrej. “The contribution of inferior colliculus activity to the auditory brainstem response (ABR) in mice.” Hearing research 341 (2016): 109–118. 27562195 10.1016/j.heares.2016.08.008

[R16] McKearney Richard M., and MacKinnon Robert C.. “Objective auditory brainstem response classification using machine learning.” International journal of audiology 58, no. 4 (2019): 224–230. 30663907 10.1080/14992027.2018.1551633

[R17] McKearney Richard M., Bell Steven L., Chesnaye Michael A., and Simpson David M.. “Auditory brainstem response detection using machine learning: a comparison with statistical detection methods.” Ear and Hearing 43, no. 3 (2022): 949–960. 34751677 10.1097/AUD.0000000000001151

[R18] Melcher Jennifer R., Knudson Inge M., Fullerton Barbara C., Guinan John J. Jr, Norris Barbara E., and Kiang Nelson YS. “Generators of the brainstem auditory evoked potential in cat. I. An experimental approach to their identification.” Hearing research 93, no. 1–2 (1996): 1–27. 8735066 10.1016/0378-5955(95)00178-6

[R19] Møller A. R., & Jannetta P. J. (1985). Neural generators of the auditory brainstem response. The auditory brainstem response, 13–31.

[R20] Pandarinath Chethan, O’Shea Daniel J., Collins Jasmine, Jozefowicz Rafal, Stavisky Sergey D., Kao Jonathan C., Trautmann Eric M. “Inferring single-trial neural population dynamics using sequential auto-encoders.” Nature methods 15, no. 10 (2018): 805–815. 30224673 10.1038/s41592-018-0109-9 PMC6380887

[R21] Radziwon Kelly E., June Kristie M., Stolzberg Daniel J., Xu-Friedman Matthew A., Salvi Richard J., and Dent Micheal L.. “Behaviorally measured audiograms and gap detection thresholds in CBA/CaJ mice.” Journal of Comparative Physiology A 195 (2009): 961–969. 10.1007/s00359-009-0472-1 PMC2813807 19756650

[R22] Rüttiger Lukas, Zimmermann Ulrike, and Knipper Marlies. “Biomarkers for hearing dysfunction: facts and outlook.” Orl 79, no. 1–2 (2017): 93–111. 28231578 10.1159/000455705

[R23] Schrode Katrina M., Dent Micheal L., and Lauer Amanda M.. “Sources of variability in auditory brainstem response thresholds in a mouse model of noise-induced hearing loss.” The Journal of the Acoustical Society of America 152, no. 6 (2022): 3576–3582. 36586874 10.1121/10.0016593 PMC9756347

[R24] Simpson M. I., and Prendergast Garreth. 2013. “Auditory Magnetic Evoked Responses.” In Handbook of Clinical Neurophysiology, 10:253–70. Elsevier.

[R25] “Streamlit, a Faster Way to Build and Share Data Apps,” n.d. https://streamlit.io/.

[R26] Suthakar Kirupa, and Liberman M. Charles. “A simple algorithm for objective threshold determination of auditory brainstem responses.” Hearing research 381 (2019): 107782. 31437652 10.1016/j.heares.2019.107782 PMC6726521

[R27] Takeda Toshio, Hosokawa Masanori, Takeshita Shuji, Irino Mika, Higuchi Keiichi, Matsushita Takatoshi, Tomita Yumiko “A new murine model of accelerated senescence.” Mechanisms of ageing and development 17, no. 2 (1981): 183–194. 7311623 10.1016/0047-6374(81)90084-1

[R28] Tucker J. D. “fdasrsf: Functional data analysis using the square root slope framework.” GitHub Repository. GitHub, https://github.com/jdtuck/fdasrsf_python (2021).

[R29] Wimalarathna Hasitha, Ankmnal-Veeranna Sangamanatha, Allan Chris, Agrawal Sumit K., Allen Prudence, Samarabandu Jagath, and Ladak Hanif M.. “Comparison of machine learning models to classify auditory brainstem responses recorded from children with auditory processing disorder.” Computer methods and programs in biomedicine 200 (2021): 105942. 33515845 10.1016/j.cmpb.2021.105942

[R30] Xie Lihong, Wang Menglin, Liao Ting, Tan Songhua, Sun Kai, Li Heng, Fang Qin, and Tang Anzhou. “The characterization of auditory brainstem response (ABR) waveforms: A study in tree shrews (Tupaia belangeri).” Journal of otology 13, no. 3 (2018): 85–91. 30559771 10.1016/j.joto.2018.05.004 PMC6291640

[R31] Young Allen, Cornejo Jennifer, and Spinner Alycia. “Auditory brainstem response.” In StatPearls [Internet]. StatPearls Publishing, 2023. 33231991

