# Supplementary material for: An Open-Source Deep Learning-Based GUI Toolbox For Automated Auditory Brainstem Response Analyses (ABRA)
Source: bioRxiv. 2024 Jun 20:2024.06.20.599815. Preprint. [Version 1] doi: 10.1101/2024.06.20.599815 (PMC11213013; doi:10.1101/2024.06.20.599815)
Supplement: Supplement 1 [file media-1.pdf]

Supplementary Information

Data Collection Methods

Detailed description of data collection methodology is described for each of the three labs as follows:

Manor Lab Data (Lab A)

- Anesthesia: 3 month SAMP8 (Senescence-Accelerated Mouse-Prone 8) (Takeda et al. 1981) mice were anesthetized using an intraperitoneal injection of a mixture of ketamine (90 mg/kg) and xylazine (10 mg/kg). If required to maintain anesthesia, mice received an additional quarter of the original dose as a top-up.
- Preparation: Ophthalmic ointment (Dechra) was applied to the subjects' eyes once anesthetized. Animals were placed in a soundproof chamber (IAC Acoustics, IL) to isolate them from exterior noise completely and a heating pad (Braintree Scientific Inc.) was used to maintain the temperature at 37°C.
- Electrode Placement: A recording electrode was inserted subcutaneously at the vertex and a reference electrode was placed behind the right pinna, with the ground electrode placed in the left leg.
- Sound Stimuli: Sound stimuli were presented via an MF1 Multi-field magnetic speaker (Tucker-Davis Technologies, TDT, FL) situated 10 cm from the mouse's right ear. Output stimuli were calibrated with a one-quarter-inch microphone (model PCB-378C01; PCB Piezotronics, NY) placed at the same distance from the speaker as the mouse ears would be.
- Recording: Electrophysiological signals in response to each tone stimulus were recorded for 10 ms starting at the onset of the tone. Stimuli were 5-ms tone pips (0.5 ms cos2 rise-fall) delivered at 21s with alternating stimulus polarity. Recorded electrical responses were filtered (300 Hz to 3 kHz) and averaged using BioSigRZ software (TDT, FL).
- Sound Intensity: The sound intensity level was decreased in 5 dB increments from 90 dB SPL to 10 or 20 dB SPL. At each sound level, 512 responses were averaged.

Marcotti Lab Data (Lab B)

- Anesthesia: Female C57BL/6N mice were anesthetized using intraperitoneal injection of ketamine (100 mg/Kg body weight, Fort Dodge Animal Health, Fort Dodge, USA) and xylazine (10 mg/Kg, Rompun 2%, Bayer HealthCare LLC, NY, USA).
- Preparation: Following the onset of anesthesia and the loss of the retraction reflex with a toe pinch, mice were placed onto a heat mat (37°C) in a soundproof chamber (MAC-3 acoustic chamber, IAC Acoustic, UK).

- Electrode Placement: Subdermal electrodes were placed under the skin behind the pinna of each ear (reference and ground electrode) and on the vertex of the mouse (active electrode) as previously described (Ingham et al., 2011).
- Sound Stimuli: Sound stimuli were delivered to the ear by calibrated loudspeakers (MF1-S, Multi-Field Speaker, Tucker-Davis Technologies, USA) placed 10 cm from the animal's pinna. Sound pressure was calibrated with a low-noise microphone probe system (ER10B+, Etymotic, USA).
- Recording: Experiments were performed using a customized software (Ingham et al., 2011) driving an RZ6 auditory processor (Tucker-Davis Technologies). Auditory thresholds were estimated from the resulting ABR waveform and defined as the lowest sound level (measured in decibels, dB) where any recognizable feature of the waveform was visible.
- Sound Intensity: Stimulus sound pressure levels were typically 0-95 dB SPL, presented in steps of 5 dB SPL. The ABR response signal was averaged over 256 repetitions.

Liberman Lab Data (Lab C)

- Anesthesia: 7 week C57Bl/6J mice were anesthetized using ketamine/xylazine anesthesia (Ketamine: 100 mg/kg, Xylazine: 10 mg/kg).
- Preparation: subjects were placed in a closed acoustic system.
- Electrode Placement: The response was recorded via needle electrodes inserted through the skin (vertex to ipsilateral pinna near tragus with a ground on the back near the tail).
- Sound Stimuli: Stimuli were 5-ms pips (0.5-ms rise-fall with a cos2 onset envelope, delivered at 30/sec).
- Recording: The response was amplified (10,000 X), filtered (100 Hz - 3 kHz), and averaged with an A-D board in a LabVIEW-driven data-acquisition system.
- Sound Intensity: The sound level was raised in 5 dB steps from roughly 10 dB below the threshold up to 80 dB SPL. At each sound level, 1024 responses were averaged (with stimulus polarity alternated.
